# A novel endoscopic ultrasound guided re-pancreaticogastrostomy using forward-viewing echoendoscope

**DOI:** 10.1055/a-2156-0444

**Published:** 2023-09-21

**Authors:** Kosuke Maehara, Tetsuo Tamura, Kazuki Hirano, Daisuke Hattori, Yoshiki Sato, Rikako Koyama, Tsunao Imamura

**Affiliations:** 1 Department of Gastroenterology, Toranomon Hospital, Tokyo, Japan; 2 Okinaka Memorial Institute for Medical Research, Tokyo, Japan

Recently, there has been a significant increase in the use of endoscopic ultrasound (EUS)-guided interventions, and several new EUS-guided drainage methods have been reported 1 2 3 . In the present case, we describe a novel technique for EUS-guided re-pancreaticogastrostomy using a forward-viewing echoendoscope.

A 57-year-old man who had undergone pancreaticoduodenectomy for recurrent pancreatic metastases from renal cell carcinoma 1 year ago presented with abdominal pain and increased serum amylase levels. Contrast-enhanced computed tomography showed a dilated main pancreatic duct (PD) in the remnant pancreas, which had been sutured to the stomach. The patient was diagnosed with obstructive pancreatitis due to stenosis of the pancreaticogastrostomy anastomosis. We attempted to dilate the stenosis using an endoscope and a guidewire inserted from the stomach. However, the anastomosis was found to be completely closed ( Fig. 1 ). Consequently, we performed EUS-guided re-pancreaticogastrostomy.

**Fig. 1:**
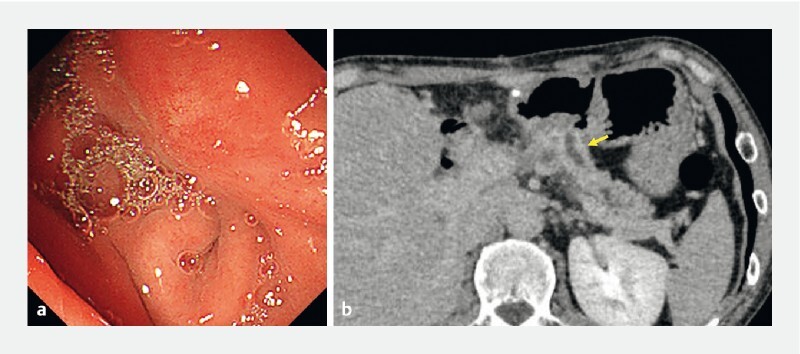
Complete anastomotic stenosis of the pancreaticogastrostomy on: **a** endoscopy; and **b** computed tomography (arrow: dilated main pancreatic duct).

Initially, a side-viewing echoendoscope (GF-UCT260; Olympus, Tokyo, Japan) was used to puncture the main PD through the stomach ( Fig. 2 ). However, it was challenging to puncture the anastomotic site and align the puncture direction with the main PD axis. Consequently, we were unable to successfully insert the plastic stent into the pancreatic tail. After 1 month, the stent had migrated to the stomach.

**Fig. 2:**
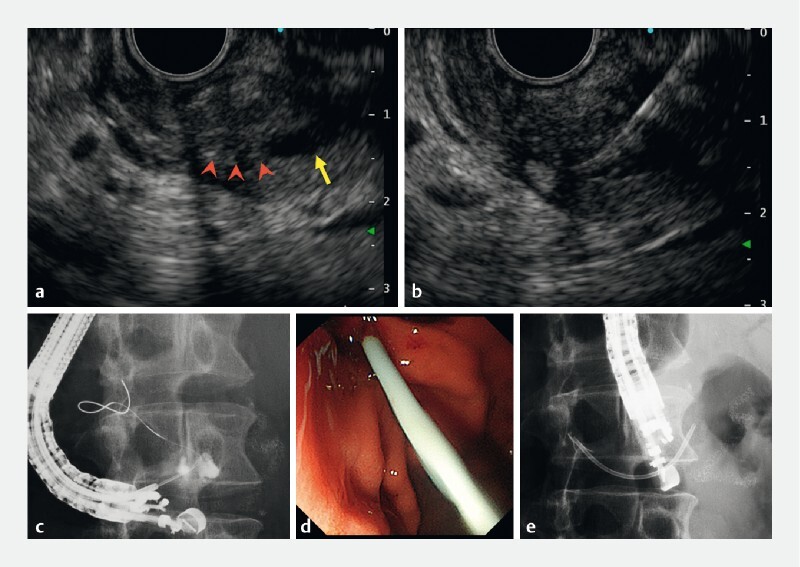
First attempt at endoscopic ultrasound (EUS)-guided re-pancreaticogastrostomy using a side-viewing echoendoscope. **a** EUS view (yellow arrow, dilated main pancreatic duct; red arrowheads, complete anastomotic stenosis). **b** Guidewire visualization on EUS. **c** Fluoroscopic view of the endoscope and guidewire. **d** Endoscopic view of unsuccessful stent deployment. **e** Fluoroscopic view of endoscope and stent.

Given the challenges encountered during the initial attempt, we reattempted the procedure using a forward-viewing echoendoscope (TGF-UC260J; Olympus) ( Video 1 ; Fig. 3 ). We successfully punctured the dilated main PD from the stomach, achieving nearly parallel axes of the echoendoscope and the main PD ( Fig. 4 ). Subsequently, we inserted a guidewire and dilator into the pancreatic tail. Finally, a plastic stent (HarmoRay 5 Fr and 5 cm; Hanaco Medical, Tokyo, Japan) was successfully placed between the stomach and the dilated main PD using the re-pancreaticogastrostomy. No adverse events were noted, and the obstructive pancreatitis was resolved. After 4 months, there was no recurrence of obstructive pancreatitis. To the best of our knowledge, this is the first case report demonstrating the use of a forward-viewing echoendoscope with EUS-guided re-pancreaticogastrostomy. The use of a forward-viewing echoendoscope appears to be safe and effective, which suggests that it is suitable for EUS-guided re-pancreaticogastrostomy.

**Video 1** Novel endoscopic ultrasound (EUS)-guided re-pancreaticogastrostomy using a forward-viewing echoendoscope.


**Fig. 3:**
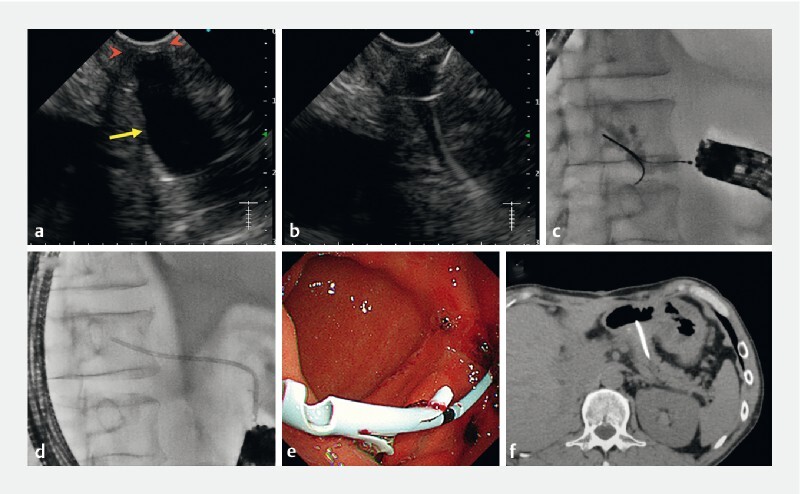
Second attempt at endoscopic ultrasound (EUS)-guided re-pancreaticogastrostomy using a forward-viewing echoendoscope. **a** EUS view (yellow arrow, dilated main pancreatic duct; red arrowheads, complete anastomotic stenosis). **b, c** Guidewire manipulation on EUS ( **b** ) and fluoroscopy ( **c** ). **d–f** Successful stent deployment seen on fluoroscopy ( **d** ), endoscopy (e), and computed tomography ( **f** ).

**Fig. 4 a:**
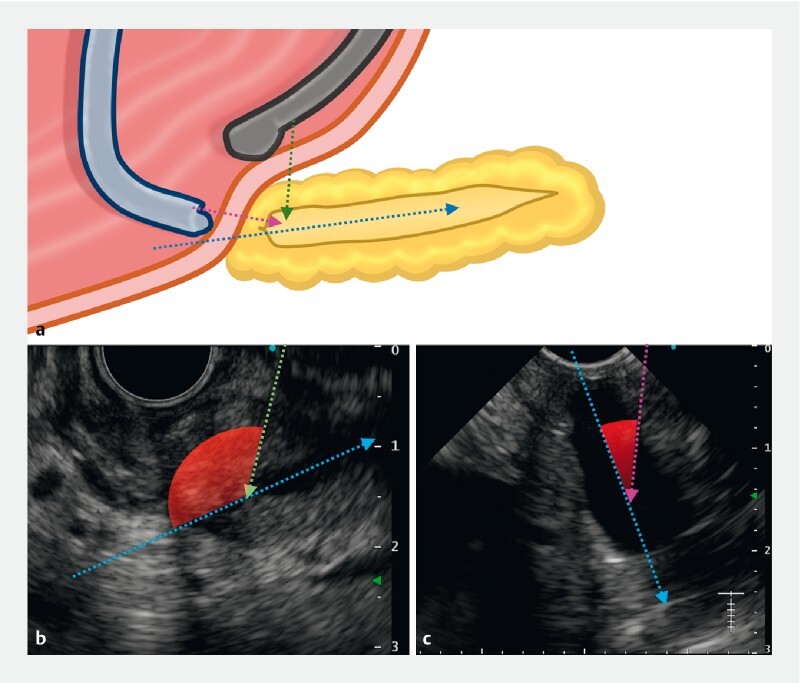
Comparison of the main pancreatic duct (PD) axis and puncture direction (green arrow, first attempt using a side-viewing echoendoscope; pink arrow, second attempt using a forward-viewing echoendoscope; blue arrow, main PD direction). **b, c** Endoscopic ultrasound (EUS) view using a side-viewing echoendoscope ( **b** ) or forward-viewing echoendoscope ( **c** ).

Endoscopy_UCTN_Code_TTT_1AS_2AD

## Notes

Endoscopy E-Videos

https://eref.thieme.de/e-videos
*E-Videos* is an open access online section of the journal *Endoscopy* , reporting on interesting cases and new techniques in gastroenterological endoscopy. All papers include a high-quality video and are published with a Creative Commons CC-BY license. Endoscopy E-Videos qualify for HINARI discounts and waivers and eligibility is automatically checked during the submission process. We grant 100% waivers to articles whose corresponding authors are based in Group A countries and 50% waivers to those who are based in Group B countries as classified by Research4Life (see: https://www.research4life.org/access/eligibility/ ). This section has its own submission website at https://mc.manuscriptcentral.com/e-videos
